# Association Between Sarcopenia Measured by Computed Tomography at the Third Lumbar Vertebra and Mortality in Inpatients with Delirium Referred to a Liaison Psychiatry Team: A Follow-Up Study

**DOI:** 10.3390/jcm14145065

**Published:** 2025-07-17

**Authors:** Miguel Alonso-Sánchez, Fernando Sebastian-Valles, María Robles-Camacho, Víctor Rodríguez-Laval, Víctor Navas-Moreno, Miguel Antonio Sampedro-Nuñez, Mónica Marazuela, Jose Luis Ayuso-Mateos, Eduardo Delgado-Parada

**Affiliations:** 1 Department of Psychiatry, Hospital Universitario de La Princesa, 28006 Madrid, Spain; chonsialonso@gmail.com (M.A.-S.); maria.robles@salud.madrid.org (M.R.-C.); joseluis.ayuso@uam.es (J.L.A.-M.); edelgadoparada@gmail.com (E.D.-P.); 2 Department of Endocrinology and Nutrition, Hospital Universitario de La Princesa, Instituto de Investigación Sanitaria de La Princesa, Universidad Autónoma de Madrid, 28006 Madrid, Spain; victornavasmoreno@gmail.com (V.N.-M.); miguelantoniosampedro@gmail.com (M.A.S.-N.); 3 Department of Radiology, Princesa Hospital, Autónoma University, 28006 Madrid, Spain; vrlaval@gmail.com; 4 Instituto de Salud Carlos III, Centro de Investigación Biomédica en Red de Salud Mental, CIBERSAM, 28029 Madrid, Spain

**Keywords:** sarcopenia, delirium, liaison psychiatry team, mortality

## Abstract

**Background and objectives**: Delirium is a prevalent disorder that is associated with morbidity and mortality in hospitalized older adults. Recent evidence highlights sarcopenia, defined by low muscle mass, as a prognostic factor of importance. This study aims to investigate the association between sarcopenia, assessed by L3-level computed tomography (CT) and clinical outcomes, particularly mortality, in inpatients with delirium managed by a liaison psychiatry team (LPT). **Methods**: This single-center, retrospective observational study included 57 consecutive patients diagnosed with delirium and referred to the LPT at a tertiary care hospital between 2021 and 2023. Patients with available abdominal CT scans were included. Sarcopenia was defined based on the presence of low muscle mass observed at the L3 level on CT imaging, following established diagnostic criteria. The analysis included demographic data, clinical history, laboratory parameters, and treatment-related variables. Cox proportional hazards models and Kaplan–Meier survival curves were utilized to evaluate the association between sarcopenia and mortality during follow-up. **Results**: Of the 57 patients included, 52.6% (*n* = 30) were sarcopenic. Sarcopenia was associated with lower albumin levels (*p* = 0.038) and higher mortality rates (56.7% vs. 33.3%). Kaplan–Meier analysis showed reduced survival in sarcopenic patients (*p* = 0.038). Cox regression identified sarcopenia as an independent predictor of mortality (HR = 2.95; 95% CI: 1.03–8.46; *p* = 0.04), alongside the Charlson comorbidity index. **Conclusions**: Sarcopenia represents a robust and independent predictor of mortality in patients with delirium. Early nutritional assessment and targeted interventions addressing sarcopenia hold the potential to improve clinical outcomes. Further prospective studies with larger sample sizes are needed to validate these findings.

## 1. Introduction

Delirium is currently defined in the ICD-11 as an acute disorder of mental state characterized by a sudden and fluctuating disturbance in consciousness and attention. It is accompanied by deficits in memory, orientation, and perception, as well as potential psychomotor disturbances and disruption of the sleep–wake cycle. The underlying cause of this condition must be attributed to the direct physiological effects of a medical condition not classified under mental, behavioral, or neurodevelopmental disorders; the direct physiological effects of a substance or medication, including withdrawal; or multiple etiological factors, including unknown etiology [1,2].

The clinical relevance of delirium lies both in its potential preventability and in its impact on health outcomes. Delirium has been consistently linked to cognitive decline, worsening of functional status, increased hospital readmissions, institutionalization, and elevated mortality rates [3,4,5]. As a potentially preventable condition, delirium underscores the need for primary prevention strategies targeting both predisposing and precipitating factors, which have become a cornerstone of healthcare quality [6,7].

There are several theories regarding the pathophysiological basis of delirium, including the neuronal aging hypothesis, the neuroinflammatory hypothesis, the oxidative stress hypothesis, the neuroendocrine hypothesis, and the circadian rhythm and melatonin dysregulation hypothesis. However, the recently proposed systems integration failure hypothesis posits that these etiological theories are complementary rather than mutually exclusive. According to this hypothesis, individuals exhibit varying degrees of physiological dysfunction, and it is this pre-existing burden that determines their baseline vulnerability to experiencing a delirium episode [8].

It is well established that the etiology of delirium is multifactorial, involving a range of predisposing factors such as pre-existing cognitive impairment, a history of prior delirium episodes, sensory deprivation, mental health disorders, and alcohol abuse. However, the presence of predisposing factors alone is insufficient for the manifestation of delirium; at least one precipitating factor must also be present. Common precipitating factors include surgical interventions, infections, polypharmacy, pre-existing brain damage, the use of psychoactive drugs, urinary catheterization, and mechanical restraints [9,10].

Delirium, malnutrition, and critical illness are known to interact in complex, mutually reinforcing ways. Acute medical stress can exacerbate nutritional decline, while pre-existing malnutrition has been linked to increased delirium risk in critically ill older adults [11,12]. Moreover, longer durations of delirium have been independently associated with greater functional deterioration, extended hospital stays, and elevated mortality in both the acute and post-discharge phases [13,14]. These interdependent pathways underscore the need for early detection and multidimensional assessment of delirium. Consequently, exploring nutritional and metabolic markers such as sarcopenia may offer valuable prognostic insight within delirium care.

The role of nutritional status as a predisposing factor for delirium remains an area of active investigation. To date, studies have established direct links between nutritional status, the onset of delirium, and mortality. However, the evaluation of nutritional status in patients with delirium has predominantly relied on indirect measures such as body mass index, muscle thickness, or the triglyceride–glucose index [15,16,17].

While these indicators provide useful insights, they are often regarded as limited in their ability to comprehensively assess nutritional status [18]. As a result, contemporary nutritional assessments extend beyond traditional anthropometric measures and include advanced techniques such as bioelectrical impedance analysis and body composition evaluation using imaging techniques like computed tomography [19,20,21,22].

Although sarcopenia has emerged as a critical prognostic factor in hospitalized populations, its study within delirium cohorts remains underdeveloped. The existing literature is often constrained by methodological limitations, including inconsistent diagnostic criteria, reliance on indirect anthropometric or biochemical markers, and brief or absent longitudinal follow-up. Notably, scoping and meta-analytic reviews in surgical and geriatric populations underscore a lack of standardization, with many studies excluding imaging-based assessments such as CT-based muscle quantification, considered the gold standard in body composition analysis [23,24,25].

Recent contributions from East Asian research groups have significantly advanced the clinical and imaging-based understanding of sarcopenia in relation to delirium, reflecting a broader global context driven by international scientific collaboration and dialogue [24,26].

We hypothesize that alterations in body composition, specifically sarcopenia, assessed through CT at the level of the third lumbar vertebra (L3), are associated with increased morbidity and mortality in inpatients with delirium referred to an LPT at a tertiary hospital, compared to those without such alterations.

## 2. Methods

This single-center, retrospective observational study included consecutive patients referred to the LPT at a tertiary care hospital. The study was conducted at [Blinded] between 2021 and 2023. Comprehensive assessments were performed for all patients, including a detailed medical history, physical examination, and mental status evaluation. Patients with available abdominal CT scans were eligible for inclusion. Exclusion criteria encompassed patients assessed by the LPT who did not develop delirium, those who did not undergo a CT scan during hospitalization, and those without post-discharge follow-up.

The study was approved by the Research Ethics Committee of Hospital Universitario de La Princesa, Madrid (study number: 5352), which granted a waiver for patient informed consent. The research adhered to the principles outlined in the Declaration of Helsinki.

### 2.1. Procedures

Delirium is one of the most common reasons for consultation in LPTs, including during the post-pandemic period [27,28]. Diagnoses made by the LPT were clinical, following the ICD-11 criteria [2]. The LPT’s role encompassed both the diagnosis and the coordinated management of delirium, primarily through pharmacological interventions in collaboration with the patient’s primary medical team. Patients referred to the LPT with delirium frequently exhibited severe clinical presentations, including marked agitation and/or treatment with multiple psychoactive medications, emphasizing the urgency and complexity of their management. The care model employed by the LPT involved proactive and immediate interventions provided on-demand, rather than within the framework of a standardized protocol.

Clinical data were collected, including age, sex, body mass index (BMI, kg/m^2^), and the Charlson comorbidity index. The ward of admission was classified as surgical, medical, or intensive care unit (ICU). Laboratory parameters at admission included hemoglobin, leukocyte count, albumin, C-reactive protein (CRP), and procalcitonin levels. Additional variables recorded were the assessment location (ICU, surgical ward, or medical ward), reason for admission (medical, surgical, or CNS-related), history of prior institutionalization, cognitive impairment, dementia, previous episodes of delirium, substance use, or alcohol consumption. Psychiatric history was categorized into none, affective disorders, anxiety, psychotic disorders, or prior mental health follow-up. Autonomy in performing basic or instrumental activities of daily living was also documented.

Pharmacological data included the use of prior medications, such as benzodiazepines (BZDs), antidepressants (ADs), antipsychotics (APs), and antiepileptic drugs (AEDs), as well as medications prescribed during admission and at discharge. Changes in prescriptions, including the initiation of new medications or deprescription during the hospital stay, were documented. Additional variables included the use of physical restraints, oxygen therapy, urinary catheterization, nasogastric tube placement, and institutionalization at discharge. Malnutrition was defined based on anthropometric criteria established by the GLIM standards: a BMI < 20 kg/m^2^ for individuals under 70 years of age and a BMI < 22 kg/m^2^ for those aged 70 years or older [29].

### 2.2. Analysis of CT Images

An experienced radiologist selected high-quality CT slides of patients at the L3 level. Images at diagnosis were used for the analysis. High contrast, unlined images, and visible surgeries were exclusion criteria. Two independent experts were trained to perform the processing of CT images with NIH ImageJ version 2.3.0 [30], whose protocol was followed [31,32]. In addition, we coded a macro script to automatically set all the Hounsfield units (HU) required to elucidate tissue areas. Then, area subtraction was executed with STATA 17.0 BE-Basic Edition statistical software (Lakeway Drive, College Station, TX, USA).

The following body composition measures were obtained: total body area, visceral fat tissue (VAT; HU = −190, −30), subcutaneous fat area (SFA; HU = −190, −30), intermuscular fat area (IMFA; HU = −190, −30), total fat area (TFA; HU = −190, −30), very-low-density muscle area (VLDM; HU = −29, −1), low-density muscle area (LDMA; HU = 0, 34), normal-density muscle area (NDMA; HU = 35, 100), high-density muscle area (HDMA; HU = 101, 150), very-high-density muscle area (VHDMA; HU = 151, 199), and total muscle area (TMA; HU = −29, 199). After the measurement step, we tested the correlation between both analyses with Spearman’s rho test. Then, we normalized the data by dividing them by the square of patients’ height in meters and, finally, we obtained the means of both measures. We only considered those with at least one year of difference from the diagnosis image. The definition of sarcopenia employed throughout this study is based on the sex-specific reference values for low L3-SMI proposed by Prado et al., which are 52.4 cm^2^/m^2^ for men and 38.5 cm^2^/m^2^ for women in obese patients (BMI ≥ 30 kg/m^2^) [21,33].

### 2.3. Statistical Analysis

Quantitative variables were expressed as medians and ranges, while qualitative variables were presented as absolute frequencies and percentages. The paired Student’s t-test was used to compare two related samples, or the Wilcoxon signed-rank test in cases of non-normal distribution. Differences between categorical variables were analyzed using the Chi-square test. The association between sarcopenia and mortality was assessed using survival analysis with the log-rank test for equality of survival curves. The relationship between continuous covariates and mortality was examined through Cox regression analysis. Potential confounding factors were evaluated based on three established criteria [34]:

− Determining whether the confounder was an independent risk factor for the outcome (mortality), assessed through significant associations between the factor and the outcome in the non-sarcopenic group.
− Assessing the association of the confounder with the exposure (sarcopenia) using mean and proportion comparisons.
− Ensuring that the confounder was not a consequence of the exposure (sarcopenia).

A multivariable Cox regression model, adjusted for identified confounders, was subsequently constructed to analyze the association between sarcopenia and mortality in patients with delirium.

Statistical analyses were performed using STATA 17.0 Basic Edition (Lakeway Drive, Austin, TX, USA). A *p*-value < 0.05 was considered statistically significant.

## 3. Results

### 3.1. Sample Characteristics

**Figure 1:**
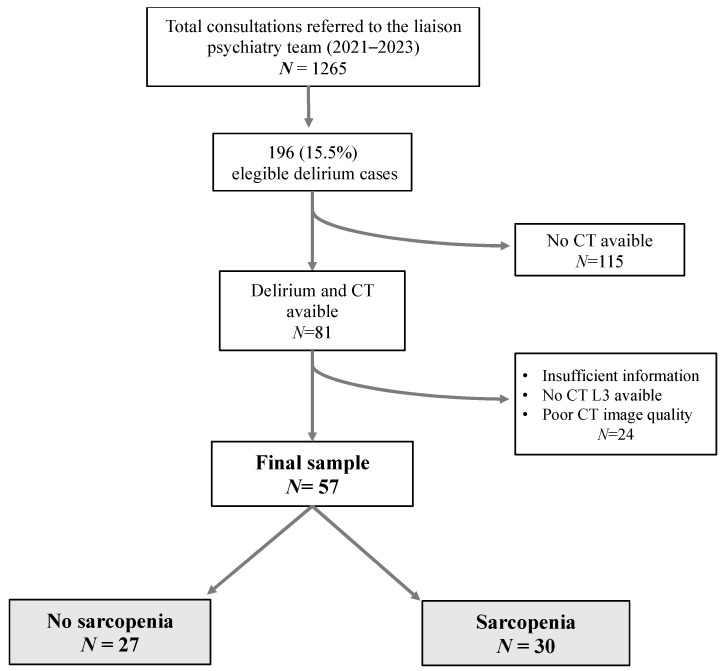
Diagram flowchart.

A total of 57 patients were included in this study. The population was divided into two groups based on the presence of sarcopenia. Among the total sample, 30 patients (52.6%) were identified as having sarcopenia, while 27 patients (47.4%) were classified as non-sarcopenic (Figure 1).

This flowchart illustrates the participant selection process for a study evaluating the association between sarcopenia and clinical outcomes. The initial cohort underwent screening, and exclusions were applied based on predefined criteria, such as insufficient clinical data or missing sarcopenia assessments. The final sample comprised two groups: participants without sarcopenia (*n* = 27) and participants with sarcopenia (*n* = 30).

The mean age of the total population was 71.1 ± 17.1 years, with no significant differences between the groups: 68.2 ± 18.3 years in the non-sarcopenic group and 73.6 ± 15.9 years in the sarcopenic group. Regarding sex, 42.1% of the patients were women, with no significant differences observed between the groups: 44.5% in the non-sarcopenic group and 40.0% in the sarcopenic group.

Regarding the Charlson index, no relevant differences were observed between the groups. The median value was 4 (3–6) in the total population, 4 (3–5) in patients without sarcopenia, and 4 (3–7) in those with sarcopenia. Hemoglobin levels were comparable across both groups, with a mean value of 10.1 ± 2.3 g/dL in the total population. In terms of assessment location, most evaluations were conducted in the intensive care unit (ICU) (45.6%), followed by the medical ward (29.8%) and the surgical ward (24.6%). Among patients without sarcopenia, 48.2% were evaluated in the ICU, 25.9% in the medical ward, and 25.9% in the surgical ward, while in patients with sarcopenia, these proportions were 43.3%, 33.3%, and 23.3%, respectively.

The reasons for admission showed significant differences between the groups (*p* = 0.024). In the total population, the most common reasons for admission were medical (42.1%) and surgical (42.1%), followed by causes related to the central nervous system (CNS; 15.8%). Among patients without sarcopenia, 37.0% were admitted for medical reasons, 33.3% for surgical reasons, and 29.6% for CNS-related causes. Conversely, in the sarcopenia group, medical admissions were more frequent (46.7%), followed by surgical admissions (50.0%), while only 3.3% were admitted for CNS-related causes.

The presence of cognitive impairment or dementia was more prevalent in patients with sarcopenia (26.7%) compared to those without sarcopenia (7.4%), although this difference did not reach statistical significance. Similarly, no differences were observed in the proportion of patients with prior episodes of delirium between the sarcopenia group (10.0%) and the non-sarcopenia group (7.4%).

### 3.2. Variables Associated with Sarcopenia

The BMI differed between the groups, with lower values observed in patients with sarcopenia (23.9 ± 1.0 kg/m^2^) compared to those without sarcopenia (27.0 ± 1.1 kg/m^2^). Regarding urea levels, although statistical significance was not reached (*p* = 0.126), patients with sarcopenia demonstrated higher levels (69.0 ± 51.8 mg/dL) compared to those without sarcopenia (46.0 ± 30.3 mg/dL). Albumin levels showed significant differences between the groups, with lower levels observed in patients with sarcopenia (3.36 ± 0.52 g/dL) compared to those without sarcopenia (3.60 ± 0.62 g/dL). See Table 1 for further details.

### 3.3. Sarcopenia and Mortality: Other Variables Associated with Mortality and Multivariable Analysis

**Figure 2:**
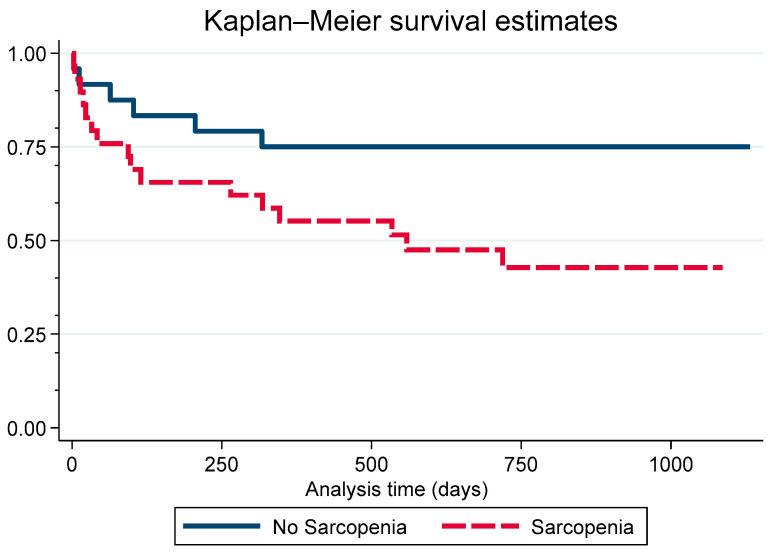
Third lumbar vertebra computed tomography images in subjects with and without sarcopenia.

**Figure 3:**
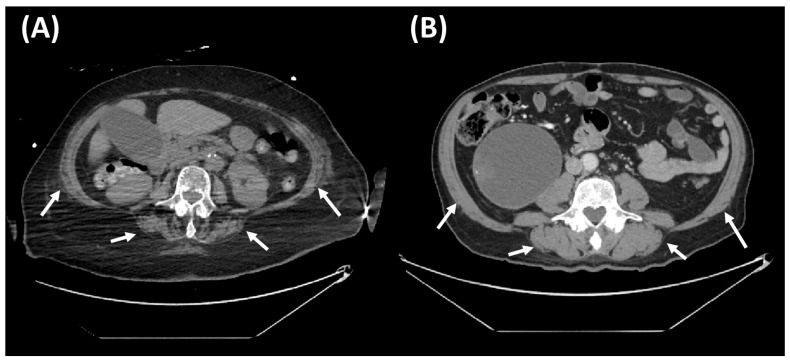
Sarcopenia and mortality: univariate analysis. (**A**): Abdominal imaging at the L3 vertebral level of an 88-year-old female patient with sarcopenia and a body mass index (BMI) of 29.9 kg/m^2^, who succumbed during follow-up. (**B**): Abdominal imaging at the L3 vertebral level of an 85-year-old male patient without sarcopenia and a BMI of 25.6 kg/m^2^, who survived during follow-up.

Among the 27 patients without sarcopenia, 9 (33.3%) died during follow-up, whereas in the sarcopenia group (*n* = 30), mortality occurred in 17 cases (56.7%). Survival analysis using the log-rank test on Kaplan–Meier curves demonstrated a significant difference between the groups (*p* = 0.038) after a median follow-up of 48.6 months (interquartile range: 7.8–70.5 months) (Figure 2). In univariate Cox regression analysis, sarcopenia was associated with an increased risk of mortality, with a hazard ratio (HR) of 1.96 (95% CI: 1.02–3.73; *p* = 0.041), compared to patients without sarcopenia. Representative imaging examples from subjects with and without sarcopenia are shown in Figure 3.

Survival analysis using Kaplan–Meier curves demonstrated lower survival rates in the sarcopenia group compared to the non-sarcopenia group. The log-rank test revealed a statistically significant difference between the survival curves (*p* = 0.03). Cox regression analysis indicated a hazard ratio (HR) of 2.61 (95% CI: 1.02–6.70; *p* = 0.045). The survival curve for patients with sarcopenia showed a sharp decline within the first 250 days, with survival rates falling to 60–70% during this period. In contrast, patients without sarcopenia maintained a survival rate of 75% over the same time frame. By 1000 days of follow-up, survival in the sarcopenia group had decreased further to 50%, whereas survival in the non-sarcopenia group remained less affected and stayed around 75%.

The association between the use of different drug classes—ADs, BZDs, APs, and AEDs—at three time points (prior to admission, during hospitalization, and at discharge) and the risk of mortality was analyzed using Cox regression models. The only significant association identified was the use of antidepressants at discharge, which was linked to a reduced risk of mortality (HR = 0.294; 95% CI: 0.090–0.963; *p* = 0.043).

A range of clinical, functional, medical procedure, and biomarker factors were analyzed in relation to mortality risk using Cox regression models. Among the factors evaluated, the Charlson index at admission was significantly associated with a higher risk of mortality (HR = 1.383; 95% CI: 1.176–1.626; *p* < 0.001). Similarly, patient assessment in the medical ward, compared to the ICU, was associated with an increased risk of mortality (HR = 2.930; 95% CI: 1.153–7.446; *p* = 0.024).

Conversely, the number of evaluations conducted by the LPT during hospitalization was associated with a reduced risk of mortality (HR = 0.871; 95% CI: 0.771–0.984; *p* = 0.026). Among the biomarkers analyzed, procalcitonin was the only parameter significantly associated with an increased risk of mortality (HR = 1.386; 95% CI: 1.050–1.830; *p* = 0.021).

Factors such as institutionalization at discharge, functional dependence, immobilization, oxygen therapy, urinary catheterization, nasogastric tube placement, sodium levels, albumin levels, and C-reactive protein levels, as well as clinical history variables including cognitive impairment, prior delirium, substance use, and alcohol consumption, did not demonstrate statistically significant associations with mortality risk in this analysis (see Supplementary Material Table S1).

**Figure 4:**
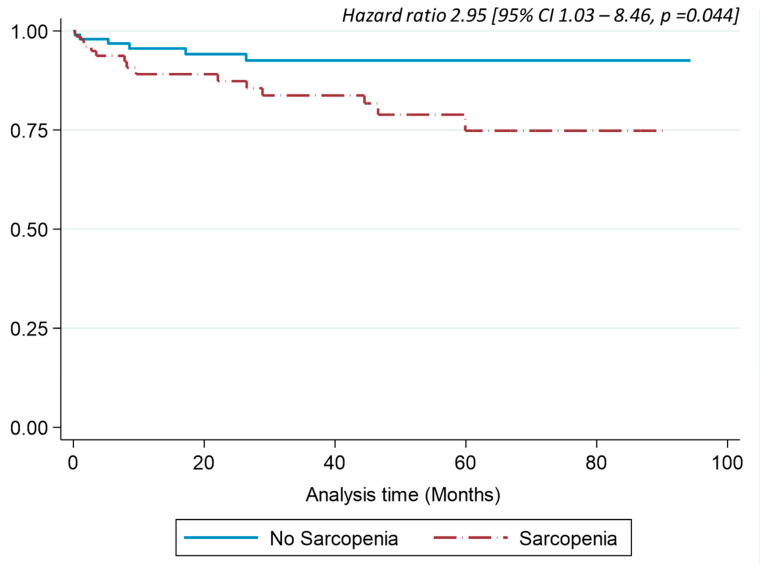
Sarcopenia and mortality: multivariable analysis.

A multivariable survival analysis using Kaplan–Meier curves and Cox regression models demonstrated that sarcopenia is independently associated with an increased risk of mortality. After adjusting for potential confounders—including BMI, GLIM criteria for malnutrition, procalcitonin levels, albumin levels, and the Charlson comorbidity index at admission—sarcopenia remained a significant predictor of mortality, with an HR of 2.95 (95% CI: 1.03–8.46; *p* = 0.04) (Figure 4). Furthermore, procalcitonin levels (HR = 1.12; 95% CI: 1.02–1.22; *p* = 0.01) and the Charlson comorbidity index (HR = 1.23; 95% CI: 1.02–1.49; *p* = 0.03) were also identified as significant predictors of increased mortality risk in patients with sarcopenia and delirium.

The multivariable survival analysis using Kaplan–Meier curves and Cox regression models demonstrated that sarcopenia is independently associated with an increased risk of mortality. During the first 40 months of follow-up, survival in the sarcopenia group decreased to approximately 80–90%, while survival in the non-sarcopenia group remained close to 100%. As the follow-up period progressed, the gap between the two groups widened further. By 80 months, survival in the sarcopenia group had declined to 75%, whereas survival in the non-sarcopenia group remained above 90%. Finally, at 100 months, cumulative survival in the sarcopenia group continued to decline, reaching levels near 70%, while survival in the non-sarcopenia group stayed above 85%.

## 4. Discussion

This study set out to explore the potential relationship between body composition as assessed through L3-level CT imaging and prognosis in hospitalized patients with delirium evaluated by an LPT in a tertiary care setting. To our knowledge, no prior studies have specifically examined this association using CT-based muscle quantification, beyond traditional indirect measures such as body mass index, the triglyceride–glucose index, or the body arm index. While these surrogate markers may offer preliminary insights, they have limited capacity to comprehensively capture nutritional status, particularly in light of current European consensus definitions of sarcopenia [35]. According to this consensus, L3-level CT imaging offers practical and precise measurements of body composition, showing strong correlations with whole-body muscle mass [36]. Additionally, this imaging technique has been employed to detect low muscle mass even in individuals with normal or elevated body weight and has demonstrated prognostic value in various clinical contexts [37,38,39].

This study appears to be the first to examine the association between nutritional status as assessed via L3-level CT imaging and mortality in patients with delirium managed by an LPT. Our findings suggest that sarcopenia may be a relevant prognostic indicator of medium-term mortality in this population, even after adjusting for other clinical variables such as the Charlson comorbidity index, the body mass index, and albumin and procalcitonin levels. While these results are exploratory and limited by sample size, they raise the possibility that early nutritional assessment, including evaluation for sarcopenia, could complement existing strategies aimed at mitigating poor outcomes in patients at risk for delirium. Furthermore, the observed association between a higher number of visits by the LPT and reduced mortality may reflect the potential benefits of specialized psychiatric support in the management of delirium. Their involvement could contribute to earlier detection and timely intervention, although further research is needed to clarify the mechanisms underlying this relationship [40].

Another notable observation was the higher mortality rate among patients managed in medical wards compared to those admitted to the ICU. This difference may reflect admission policies and clinical decision-making processes, where patients with poorer prognoses or advanced comorbidities are more likely to remain in medical wards. However, this association should be interpreted with caution, as it may be influenced by unmeasured factors such as treatment limitations, pre-existing advance directives, or institutional variability in triage practices [41].

Lastly, the observed association between antidepressant use at discharge and reduced mortality should be interpreted cautiously. Rather than implying a direct pharmacological effect, this finding may reflect differences in patient profiles, such as better functional status, greater psychosocial support, or closer clinical follow-up among those for whom antidepressants were prescribed after a delirium episode. Given the observational nature of our study, further investigation is needed to clarify the potential mediators of this relationship. Although the prior literature suggested a possible association between cognitive impairment and either sarcopenia or mortality [42,43], we did not observe such a relationship in our cohort. This finding may be partially explained by the specific clinical characteristics of patients referred to the LPT, who often present with psychiatric comorbidities and exposure to psychotropic medications, yet may retain relatively preserved functional and cognitive status prior to hospital admission. It is also possible that acute stressors or precipitating events, rather than baseline cognitive decline, play a more prominent role in delirium onset and outcomes within this subgroup. Nonetheless, these interpretations remain speculative and highlight the need for further prospective research using comprehensive neurocognitive assessments in this population [44,45].

While this study provides exploratory insight into the prognostic value of body composition in patients with delirium, several limitations must be acknowledged. First, the sample derives exclusively from hospitalized individuals referred to the LPT, limiting generalizability to community-dwelling older adults. Muscle wasting during acute illness may not reflect trajectories observed in outpatient or subacute care settings. Second, comorbid conditions such as infections, malignancies, or endocrine disorders could act as confounding variables, potentially influencing both sarcopenia and mortality. Third, the retrospective observational design precludes any inference of causality and may introduce selection or information bias. Lastly, the small sample size limits statistical power and increases the risk of false-negative or unstable associations. These limitations emphasize the exploratory nature of our study and suggest that its main contribution lies in formulating a novel hypothesis within a clinical population that has been scarcely examined in relation to body composition. Rather than offering definitive conclusions, our findings aim to prompt further research into the potential prognostic role of sarcopenia in patients with delirium, and to encourage a more integrated approach to the nutritional and functional assessment of this vulnerable group.

Sarcopenia, as assessed by CT imaging at the third lumbar vertebra, emerged as a potentially important prognostic marker of mortality in hospitalized patients with delirium evaluated by the LPT. However, this association must be interpreted cautiously and within the context of the methodological limitations described above. Future research should aim to replicate these findings in larger, multicenter cohorts using prospective designs, incorporate standardized diagnostic protocols, and extend follow-up to capture post-discharge outcomes. Additionally, further studies are needed to better characterize the complex interplay between chronic illness, nutritional status, sarcopenia, and delirium in older adults.

## Figures and Tables

**Table 1:** Baseline characteristics of the sample in relation to the presence of sarcopenia.

Variables	Obs N = 57	No Sarcopenia N= 27 (47.37%)	Sarcopenia N= 30 (52.6%)	*p*-Value
Age (years)	71.1 ± 17.1 ^1^	68.2 ± 18.3 ^1^	73.6 ± 15.9 ^1^	0.106
Sex (female)	24 (42.1%)	12 (44.5%)	12 (40.0%)	0.734
**BMI (Kg/m^2^)**	**25.4 ± 5.7 ^1^**	**27.0 ± 1.1 ^1^**	**23.9 ± 1.0 ^1^**	**0.039**
Charlson scale	4 (3–6) ^2^	4 (3–5)	4 (3–7)	0.438
Hemoglobin	10.1 ± 2.3 ^1^	10.1± 2.0 ^1^	10.1 ± 2.5 ^1^	0.930
Urea	58.2 ± 44.1 ^1^	46.0 ± 30.3 ^1^	69.0 ± 51.8 ^1^	0.126
**Albumin**	**3.48 ± 0.58 ^1^**	**3.60 ± 0.62 ^1^**	**3.36 ± 0.52 ^1^**	**0.038**
eGFR (mL/min)	81.4 ± 46.7 ^1^	78.5 ± 37.9 ^1^	83.9 ± 53.6 ^1^	0.851
Sodium	139 ± 4 ^1^	139 ± 3 ^1^	139 ± 5 ^1^	0.704
RCP ng/mL	6.2 (1.7–14.4) ^2^	5.2 (2.8–10.8) ^2^	8.1 (1.5–19.7) ^2^	0.364
Procalcitonin	0.27 (0.06–0.45) ^2^	0.27 (0.06–0.52) ^2^	0.27 (0.07–0.45) ^2^	0.510

BMI: body mass index; CRP (ng/mL): C-reactive protein (ng/mL); eGFR (mL/min): estimated glomerular filtration rate (mL/min). ^1^. Mean ± standard deviation. ^2^. Median (interquartile range: P25–P75).

## Data Availability

The datasets generated during and/or analyzed during the current study are available from the corresponding author on reasonable request.

## Supplementary Materials

The following supporting information can be downloaded at: https://www.mdpi.com/article/10.3390/jcm14145065/s1, Table S1: Cox Regression Analysis Results.
