# Supplementary material for: Association Between Sarcopenia Measured by Computed Tomography at the Third Lumbar Vertebra and Mortality in Inpatients with Delirium Referred to a Liaison Psychiatry Team: A Follow-Up Study
Source: J Clin Med. 2025 Jul 17;14(14):5065. doi: 10.3390/jcm14145065 (PMC12296102; doi:10.3390/jcm14145065)
Supplement: Supplementary file 1 [file jcm-14-05065-s001.zip › jcm-3745812-supplementary.pdf]

Supplementary Table: Cox Regression Analysis Results

Variable	HR	95% CI	p-value
Sarcopenia (yes vs no)	2.61	1.02–6.70	0.045
Antidepressants at discharge	0.294	0.090–0.963	0.043
Charlson index at admission	1.383	1.176–1.626	<0.001
Medical ward vs ICU	2.93	1.153–7.446	0.024
No. of LPT evaluations during hospitalization	0.871	0.771–0.984	0.026
Procalcitonin (continuous)	1.386	1.050–1.830	0.021
Institutionalization at discharge	1.15	0.80–1.64	0.45
Functional dependence	1.08	0.79–1.48	0.62
Immobilization	1.2	0.88–1.63	0.29
Oxygen therapy	0.95	0.70–1.29	0.75
Urea (mg/dL)	1.12	0.82–1.52	0.52
Nasogastric tube placement	1.05	0.76–1.46	0.64
Sodium levels	0.99	0.73–1.35	0.93
Albumin levels	0.89	0.66–1.21	0.47
C-reactive protein levels	1.07	0.81–1.42	0.6
Cognitive impairment	1.1	0.79–1.53	0.49
Prior delirium	1.13	0.84–1.51	0.43
Substance use	1.04	0.71–1.52	0.87
Alcohol consumption	0.98	0.70–1.37	0.89
